# Multi-view and multi-scale behavior recognition algorithm based on attention mechanism

**DOI:** 10.3389/fnbot.2023.1276208

**Published:** 2023-09-26

**Authors:** Di Zhang, Chen Chen, Fa Tan, Beibei Qian, Wei Li, Xuan He, Susan Lei

**Affiliations:** ^1^Department of Telecommunications, Xi'an Jiaotong University, Xi'an, China; ^2^School of Information Engineering, Xi'an Eurasia University, Xi'an, China

**Keywords:** teaching behavior analysis, attention mechanism, intra-class differential representation learning, human behavior, behavior recognition

## Abstract

Human behavior recognition plays a crucial role in the field of smart education. It offers a nuanced understanding of teaching and learning dynamics by revealing the behaviors of both teachers and students. In this study, to address the exigencies of teaching behavior analysis in smart education, we first constructed a teaching behavior analysis dataset called EuClass. EuClass contains 13 types of teacher/student behavior categories and provides multi-view, multi-scale video data for the research and practical applications of teacher/student behavior recognition. We also provide a teaching behavior analysis network containing an attention-based network and an intra-class differential representation learning module. The attention mechanism uses a two-level attention module encompassing spatial and channel dimensions. The intra-class differential representation learning module utilized a unified loss function to reduce the distance between features. Experiments conducted on the EuClass dataset and a widely used action/gesture recognition dataset, IsoGD, demonstrate the effectiveness of our method in comparison to current state-of-the-art methods, with the recognition accuracy increased by 1–2% on average.

## 1. Introduction

Human behavior recognition technology encompasses the analysis and identification of human body postures, movements, and specific actions ultimately leading to the recognition of human behaviors. Currently, human-body recognition techniques are applied in smart education, smart security, human-computer interaction, and health monitoring, among other fields. Various behavior recognition methods have emerged as technologies have continued to advance. Commonly employed human behavior recognition methods include machine learning methods based on feature extraction and classifier training. Early on, probabilistic and statistical methods (Yamato et al., 1992; Natarajan et al., 2010; Shi et al., 2011) were often utilized for behavior identification. Many innovative methods have since surfaced with the advent of deep learning, such as convolutional neural networks (CNNs) and recurrent neural networks (RNNs) (Xu et al., 2014; Muhammad et al., 2021; Shi et al., 2023a,b; Tian et al., 2023; Zhaowei et al., 2023), which have been extended to teaching behavior analysis tasks (Li et al., 2021; Lin et al., 2021; Xie et al., 2021; Zhao et al., 2021; Gu and Li, 2022; Guo, 2022).

Despite the successes of these methods, certain challenges remain. Many approaches rely on datasets for training and testing purposes, but most widely used datasets are confined to controlled laboratory settings. These settings have limited environmental variability, allow for only negligible changes in perspective and scale, and do not reflect the difficulty of discerning behaviors from intricate backgrounds or low-light conditions. The environmental conditions within which actual teaching and learning behaviors are performed often entail greater complexity and dynamic shifts than a laboratory. There is a need to curate datasets that mirror these real-world fluctuations, more closely approximating authentic classroom conditions to ensure optimal recognition outcomes.

Furthermore, given that classroom teaching behavior constitutes a continuous sequence of actions, it is imperative not only to comprehend spatial attributes within individual frames of video data but also to capture temporal patterns. In this context, employing the attention mechanism to grasp both temporal and spatial fluctuations within actions is crucial. Additionally, disparities exist among distinct feature maps within the same layer of the network architecture. Certain feature maps may encapsulate more informative content while others do not. Accordingly, it is essential to account for the correlation between different feature maps to enhance the efficacy of feature representation.

It is worth noting that there is a substantial difference between classroom teaching behavior and general behavior. Classroom settings inherently impose constraints, so teaching behavior analyses primarily focus on the upper body while excluding movements of the lower body and other regions. Consequently, developing a unified feature representation model is essential to mitigate feature differences arising from differing distances and viewing angles within the same class of samples, as well as to prevent interference from environmental variables.

We constructed a dedicated teaching behavior dataset in this study in an effort to resolve the problems described above. We also developed a teaching behavior analysis network which contains an attention-based network and an intra-class differential representation learning module. The attention mechanism uses a two-level attention module spanning spatial and channel dimensions, while the intra-class differential representation learning module uses a unified loss function to reduce the distance between features.

The contributions of this work can be summarized as follows:

1. We compile a classroom teaching behavior dataset that encompasses both student and teacher actions. A comprehensive set of classroom teaching data is curated to meticulously capture intricate classroom behavior dynamics, through the utilization of different perspectives and scales. The dataset will be publicly available.
2. A multi-view and multi-scale behavior recognition method based on the attention mechanism is proposed. This method comprises an attention mechanism module coupled with an intra-class differential representation learning module. The modules collectively mitigate the impact of environmental variables on recognition outcomes, enhancing the model's adaptability to real-world scenarios.
3. The proposed approach is empirically validated through experiments on our EuClass dataset and the widely used public IsoGD dataset. The results indicate that our method outperforms other state-of-the-art methods.

The rest of the paper is organized as follows. Section 2 reviews previous work relevant to the present study. Section 3 outlines the proposed method in detail. Section 4 introduces the new collected dataset and Section 5 presents the evaluation results. Section 6 provides a summary and conclusion.

## 2. Related works

### 2.1. Behavior recognition method

In recent years, behavior recognition has emerged as a pivotal subject in the field of computer vision research. There have been numerous studies on classroom teaching behavior recognition. Li et al. (2021), for instance, used a support vector machine (SVM) and CNN to obtain characteristic data for classroom teaching behavior, achieving heterogeneous support vector samples for online learning behavior. They significantly enhanced recognition accuracy, with an evaluation error of 1.9% with 20 iterations.

In another study, Xie et al. (2021) used feature engineering in conjunction with k-means clustering (KMC) on classroom surveillance videos to perform cluster analysis on different student groups, effectively identifying abnormal behavior among college students. Lin et al. (2021) used continuous frames captured by classroom cameras as input images for their system coupled with skeleton data collected from the OpenPose framework. Feature extraction was performed to represent feature vectors of human poses, including normalized joint positions, joint distances, and bone angles to ultimately identify student behaviors. The approach exhibited a 15.15% increase in average precision and a 12.15% surge in average recall compared to skeleton-based methodologies.

Guo (2022) used a database containing 2,500 images of five behaviors (e.g., raising hands, sitting up, writing, sleeping, and mobile phone usage) for object detection; they extracted frames from classroom screen recording videos using the OpenCV library, then transformed the virtual network into MobileNet to complete the fusion function. Compared with the traditional single-shot detector (SSD) method, their model more accurately recognized small objects with no significant decrease in recognition speed. Gu and Li (2022) proposed a fast target detection method based on FFmpeg CODEC and MHI-HOG joint features, establishing a behavior recognition model through a joint back propagation (BP) neural network-SVM joint classifier based on a lookup table. Their classifiers effectively facilitated the establishment of smart classrooms. Zhao et al. (2021) and other researchers pioneered the concept of a “teacher set” within extensive teaching videos. Building upon this concept, they developed a teacher-set identification and extraction algorithm, the teacher-set IE algorithm. An advanced 3D bilinear pooling-based behavior recognition network (3D BP-TBR) was proposed to categorize teacher behaviors. Experimental results demonstrated the superior performance of 3D BP-TBR across public and self-built datasets (TAD-08).

### 2.2. Attention mechanism

The attention mechanism is based on the findings of cognitive researchers. The human brain prioritizes important information and disregards unimportant information during information processing. This concept has been applied to computer vision technology, where its incorporation has bolstered the performance of network models, making it a popular tool in many computer vision tasks (Li et al., 2019a; Chen et al., 2022). In the context of behavior recognition, the presence of occluders and background interference can affect recognition accuracy. Some researchers have used the attention mechanism to guide their networks' focus on the behavior being analyzed.

To enhance the quality of spatio-temporal features extracted from sparse skeleton data, Xia et al. (2022) introduced two attention modules: Motion-guided Channel Attention Module (MGCAM) and Spatio-Temporal Attention Module (STAM). MGCAM calculates temporal frame-level motion to establish interdependence between feature channels. STAM, conversely, orchestrates context-aware collaboration across space and time at the sequence level, extracting attention features that account for long-range dependencies. The fusion of MGCAM and STAM resulted in LAGA-Net, an architecture that extracts discriminative features by integrating local and global representations of skeleton sequences.

In the realm of video saliency detection, Xu et al. (2021) deconstructed spatio-temporal feature learning into distinct stages. They innovatively combined several attention models into each stage to concentrate on information from different representation subspaces at varying points. This approach significantly enhances the efficacy of saliency detection, resulting in improved overall performance.

### 2.3. Feature representation optimization

Feature representation optimization involves refining the way data is represented to enhance its compatibility with machine learning algorithms. This is done by transforming raw data into sets of feature vectors suitable for algorithmic input. A well-crafted feature representation assists algorithms in uncovering data patterns and regularities more effectively, thereby improving the performance and generalization ability of the model. Currently, feature representation optimization is widely applied in the fields of machine learning and deep learning.

Ding et al. (2021) introduced a method called channel multiplexing to economize memory usage and model parameters. Channel multiplexing involves using different convolution kernels to generate multiple feature maps within the same convolutional layer. The feature maps are then combined and input to the subsequent convolutional layer, imbuing the network with variations by integrating diverse depths and widths into a single convolutional layer. Gomez et al. (2017) proposed a new self-supervised learning framework, CMP, to optimize feature representation by learning the spatial relationship between different objects in videos. This method has achieved excellent performance across various vision tasks.

Xue et al. (2021) proposed a multi-objective feature selection method based on reinforcement learning, which guides the model to select optimal features according to a reward function. Their model achieved better results than traditional feature selection methods. Qian et al. (2021) introduced a multi-level feature optimization framework tailored to enhancing the generalization and temporal modeling capabilities of learned video representations. The process involves leveraging high-level features derived from original and prototype contrastive learning to construct distribution maps. These maps, in turn, guide the acquisition of low-level and mid-level features. Zotin et al. (2018) used classifiers based on fuzzy logic in order to capture all the nuances of uncertainty encountered. Versaci et al. (2022) proposed an innovative data classification procedure based on fuzzy similarity cumulations capable of classifying data by grouping them according to similarity, in a fuzzy sense, by extracting for each class a reduced set of data representing that particular class. Li et al. (2023a) leveraged the idea of information bottleneck to refine the gesture feature and avoided the influence of environmental interference like the illumination and background.

## 3. Proposed method

### 3.1. Overview

**Figure 1:**
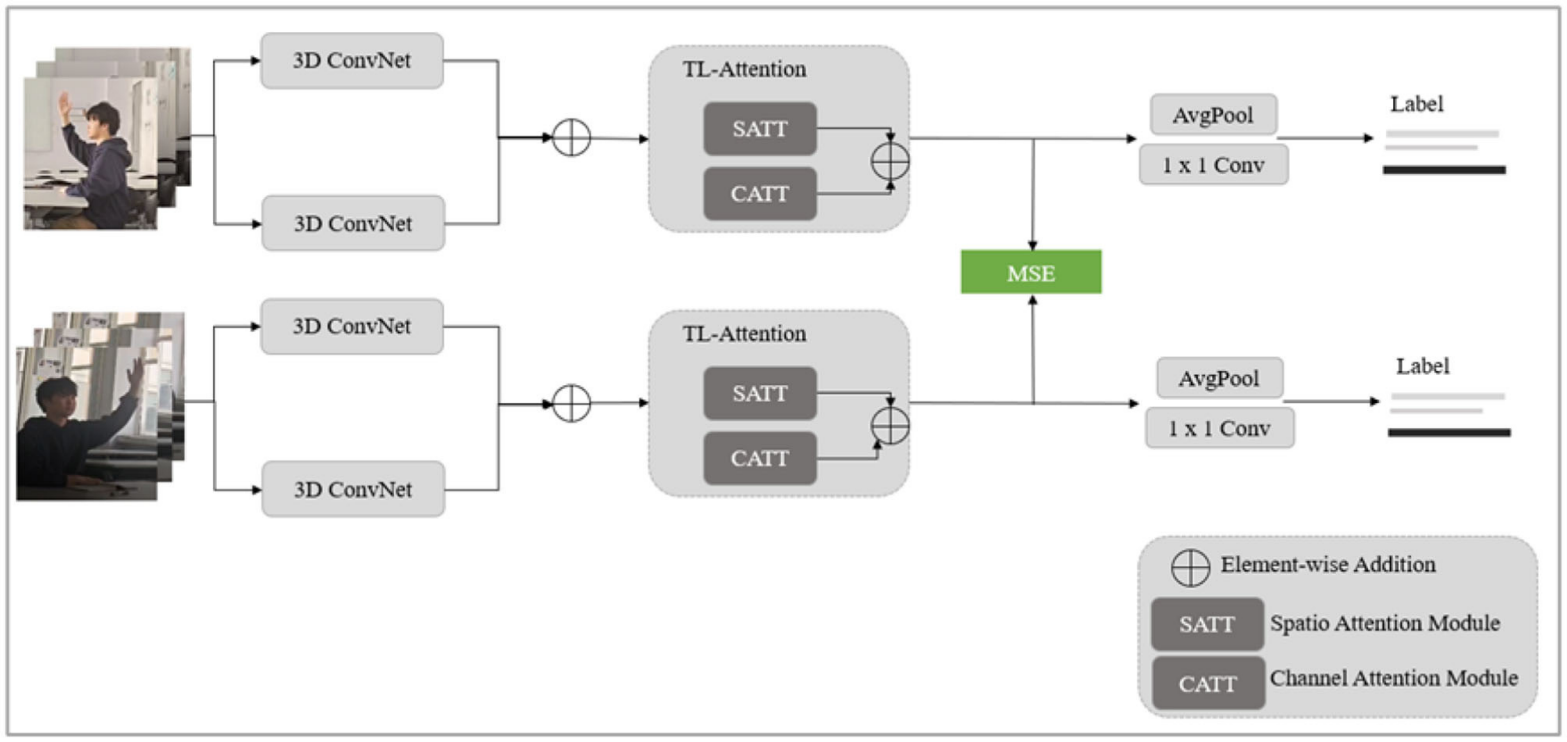
Structural diagram of multi-view and multi-scale behavior recognition method based on attention mechanism.

As shown in Figure 1, we developed a novel method for recognizing actions in classroom behavior videos. This method uses a multi-view and multi-scale framework coupled with an attention mechanism. The process begins by extracting video features using the I3D model and then implementing a two-layer attention mechanism model to focus on the spatiotemporal features of the behaviors. The spatiotemporal attention module deploys a self-attention mechanism to identify spatial location information within the human area of the video. Concurrently, the channel attention mechanism prioritizes channels that are more relevant to the recognition task and have richer information. This refinement improves the feature representation capability.

Acknowledging the variability introduced by real-world classroom settings where the camera captures students and teachers from various distances and angles we designed our model to include an intra-class differential representation learning module. This was achieved by constructing two weight-sharing networks, facilitating the learning of target representation differences across different scales and perspectives. By unifying the loss function to mitigate intra-class differences, this module assists in learning behavioral features that are robust and representable.

### 3.2. Attention mechanism

**Figure 2:**
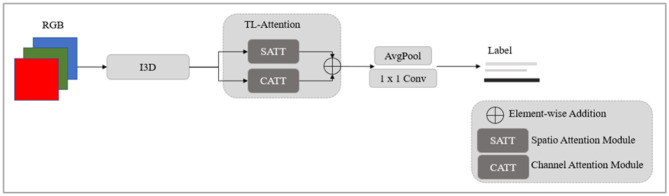
Behavior recognition based on a two-level attention mechanism.

The position and posture of the human body captured in a video change over time, and the convolution operation creates issues in the local receptive field, so establishing consistent features linked to the human body area across a temporal range is challenging. To address this, we developed a two-level spatiotemporal attention module comprised of a spatial attention module (SATT) and a channel attention module (CATT), as shown in Figure 2.

**Figure 3:**
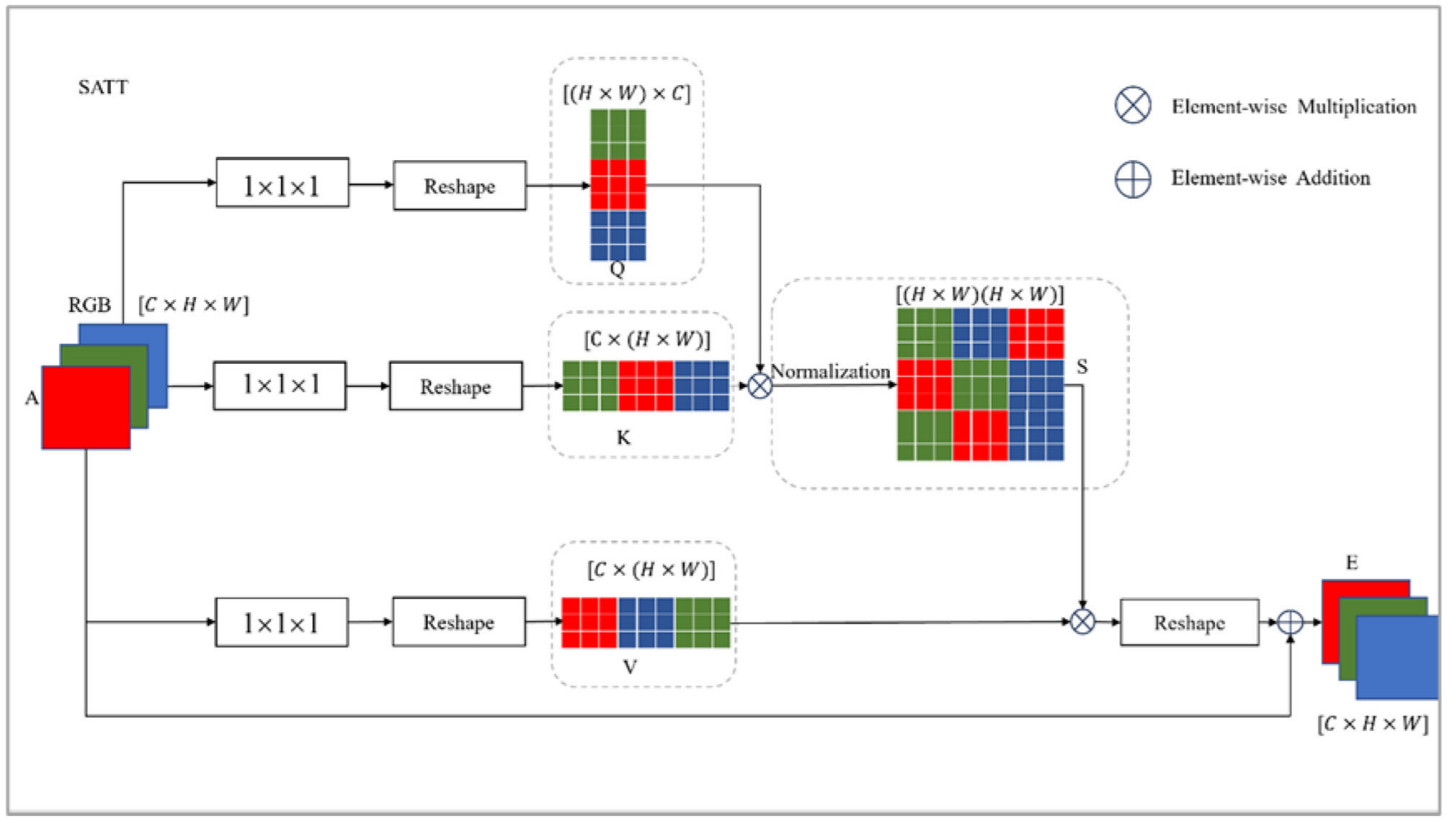
Spatial attention module structure. This module is designed to capture the relations between features in the spatial domain. It leverages a self-attention structure with the features from I3D as Q, K, and V, respectively. Then the output feature is yielded via a weighted sum.

As depicted in Figure 3, the SATT architecture captures the features of the spatial dimension, then the input features are embedded through a convolution operation. The dimension transformation operation of the matrix generates a query matrix *Q*, key matrix *K*, and value matrix in the attention mechanism *V*. Matrix multiplication is performed on the *Q* and *K* matrices to model the spatial relationship between any two positions of the feature. Softmax is then applied for normalization processing, generating a spatial distribution matrix of attention weights. The calculation process of matrix *s* is:


(1) $$s_{ij}\;=\;\frac{\exp(Q_{i}^{\;}K_{j}^{\;})}{\sum_{j=1}^{N}\exp(Q_{i}^{\;}K_{j}^{\;})}$$

where *Q* ∈ *R* ^*N*×*C*^ *, K, V* ∈ *R* ^*C*×*N*^ *(N* = *H* × *W)* represent the embedding matrix of *A*. The coefficients *H, W*, and *C* denote the height, width, and channel of the feature map; *s*_*ij*_ is an element of the spatial attention weight distribution matrix that represents the similarity between position *i* and position *j*. The output of SATT can be expressed as follows:


(2) $$E_{i}=\alpha \sum_{j=1}^{N}(s_{ij}V_{j}^{\;})+A_{i}$$

where *E* is the spatial attention result, which is multiplied by *S* and *V*, then multiplied by the learnable parameter α for element-wise summation with feature *A*. Each location in *E* is the result of a weighted and selective aggregation of features from all locations with the original features. Therefore, each location contains information from the global context.

**Figure 4:**
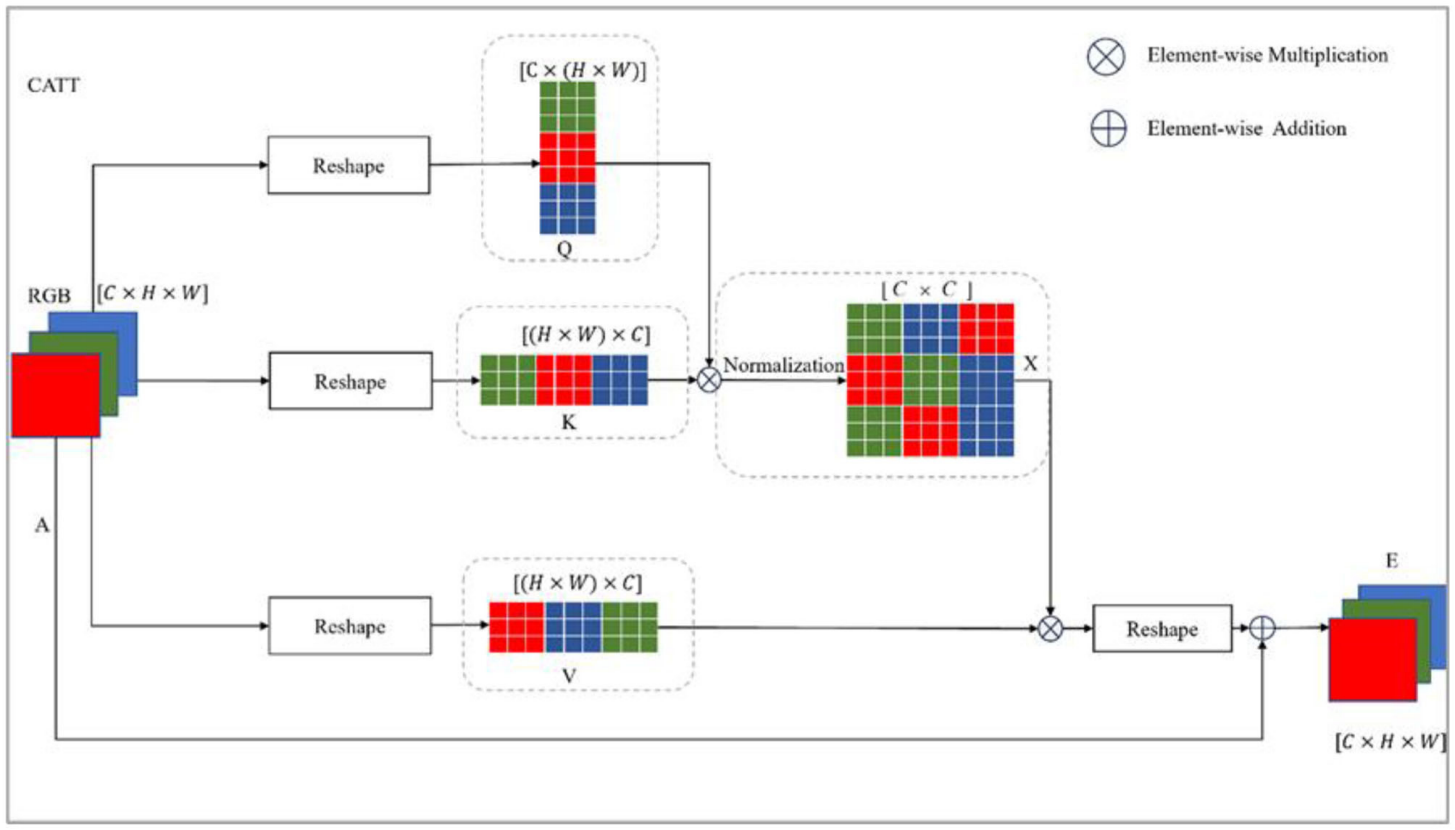
Channel attention module structure. Similar to the spatial attention module structure, it also employs a self-attention structure but learns attention from the features in different channels.

The feature map of each channel can be viewed as a high-level semantically specific response to the current task. It is associated with different semantic responses. The channel attention module explicitly models the interdependencies between channels and captures contextual information in the channel dimension. The channel attention module operates similarly to the spatial attention module, as shown in Figure 4. The input features *A* ∈ *R*^*C*×*H*×*W*^ are generated by matrix reconstruction *Q* ∈ *R*^*C*×*N*^, *K, V* ∈ *R*^*N*×*C*^, and *N* = *H* × *W*. The channel attention weight distribution matrix *X* ∈ *R*^*C*×*C*^ is then obtained by matrix multiplication of *Q* and *K* and Softmax normalization (Equations 3, 4). After multiplying matrices *X* and *V*, the result is applied to *A* and multiplied by a learnable parameter *β*.


(3) $$x_{ij}\;=\;\frac{\exp(Q_{i}^{\;}K_{j}^{\;})}{\sum_{j=1}^{C}\exp(Q_{i}^{\;}K_{j}^{\;})}$$

(4) $$E_{i}\;=\;\beta \sum_{j=1}^{C}(x_{ij}V_{j}^{\;})+A_{i}$$

Similar to SATT, after the feature map of each channel is established, the feature is processed by the spatio-temporal channel attention module. This is the result of the selective aggregation of features on all channels, which is the weighted sum of the original features. The processed feature maps capture long-term semantics among channels accordingly.

### 3.3. Differentiated representation learning for similar behaviors

**Figure 5:**
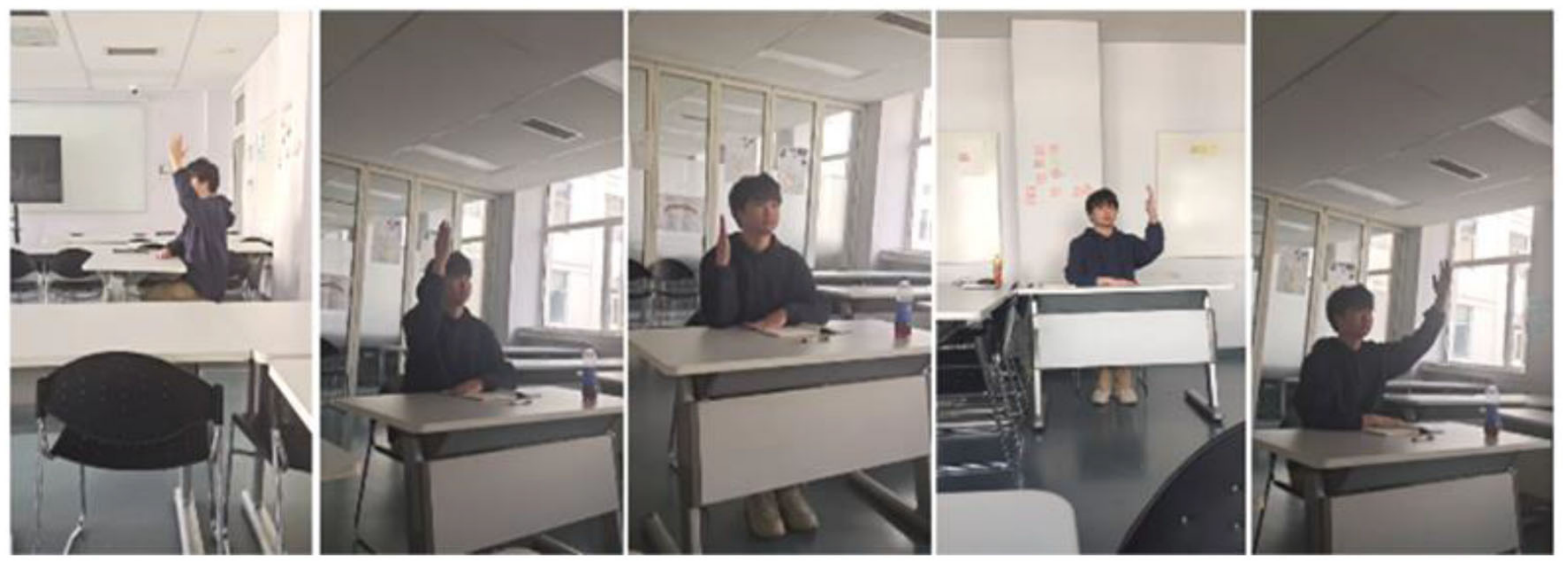
Images of different shooting angles of raising hands.

As shown in Figure 5, for the same type of classroom teaching behavior videos, the visual presentation may vary due to differences in performers' distances from the camera and varying viewing angles. This issue is prevalent in real-world classroom teaching behavior videos. Importantly, these factors unrelated to behavior recognition significantly influence the recognition algorithm's performance. This impact becomes particularly pronounced when performers are distant from the camera, making behavior identification challenging. The overemphasizing distance information can lead to overfitting problems, ultimately undermining the recognition accuracy of videos captured from long distances.

Figure 1 illustrates the proposed intra-class differential representation learning module, which was designed to learn representations for action videos within the same category. The module consists of a two-level feature learning network. One level randomly inputs a certain type of action video. After determining the input of this level, the other level makes selections according to the similarity and selects a type of video similar to it. The video with the smallest difference degree is used as the input of the intra-class differentiation representation. The similarity selection is realized by the following cosine similarity function:


(5) $$cossim(u,v)\;=\;\frac{u\;\cdot \;v}{∥u∥\cdot \;∥v∥}$$

where u and v are the vectors composed of two respective videos.

After determining the inputs for both levels, features are extracted using a shared weight network. Considering that videos of the same action type may exhibit considerable differences in low-level representations, their high-level features should remain consistent in the semantic space to ensure precise recognition outcomes. Following feature extraction, it is necessary to ensure feature consistency among differentially represented videos by employing the following similarity function (James et al., 2013):


(6) $$MSE\;=\;\frac{1}{n}\sum_{i=1}^{n}{\left({\hat{y}}_{i}-y_{i}\right)}^{2}$$

where *n*, *y*_*i*_, and ŷ_*i*_ represent the number of samples, actual value, and predicted value, respectively.

## 4. Dataset construction for classroom teaching analysis

In an analysis of classroom teaching behavior, Wen et al. (Zhang et al., 2020) constructed a new dataset called SICAU-Classroom Teaching Behavior which includes 584 images and 31,380 annotated objects. They integrated the CBAM attention mechanism module in YOLO3, enhancing the target behavior detection rate. Fu et al. (2019) constructed a classroom learning behavior dataset called ActRec-Classroom, which includes behaviors such as listening, fatigue, raising hands, and reading/writing over a total of 5,126 images. They employed OpenPose to extract human skeletal, facial, and finger key point features, then applied a CNN-based classifier to reach a behavior recognition accuracy of 92.86%.

Sun et al. (2021) collected 128 videos from different subjects across 11 different classroom settings. Their dataset consists of a detection part, a recognition part, and a subtitle part. The detection part incorporates a temporal detection data module (4,542 samples) and an action detection data module (3,343 samples), the recognition part contains 4,276 samples, and the subtitle part contains 4,296 samples. They analyzed classroom scenario characteristics and the technical intricacies of each module (task), offering baseline comparisons with mainstream datasets. Tang et al. (2022) manually constructed a classroom teaching behavior detection dataset based on real classroom videos. Their dataset includes four behavior types: listening, looking down, lying down, and standing. Fan (2023) developed a public behavioral dataset that focuses on raising hands, which is directly drawn from genuine in-class recordings.

Existing classroom teaching behavior datasets were mainly constructed based on classroom recordings. This approach has limitations in terms of data and source variety, however, making it difficult to comprehensively represent the intricate micro-behaviors taking place in actual classrooms. Furthermore, given the involvement of a significant number of teachers and students in these datasets, safeguarding their personal information is crucial to prevent any potential misuse.

Considering these concerns, we adopted a meticulous approach by closely observing classroom dynamics. We engaged a single teacher and a single student to partake in isolated classroom teaching behavior recordings. We used the recordings to formulate the EuClass dataset, which contains 1,456 video samples of 13 classroom behaviors. Different perspectives and video scales can affect the accuracy of behavior recognition, so when constructing the EuClass dataset, we captured images of teachers' teaching behaviors and students' learning behaviors from different perspectives and different scales as a targeted strategy to enhance recognition accuracy (Table 1).

**Table 1:** Basic information for the EuClass dataset.

**Superclass**	**Class**	**Description**
Sit	Sitting upright	Standing upright with feet flat on the ground
	Half-crossed legs	Resting the sole of one foot on the thigh of the other leg
	Cross-legged sitting	Crossing feet under the chair and leaning forward slightly
	Legs swinging	Feet hanging in the air, rocking back and forth
Stand	Upright	Feet flat on the ground, body upright
	Bent over	Lowering upper body toward the floor
Walk	Walking on one side	Alternating feet to move the body forward
	Pacing	Walking back and forth in a certain area at a moderate step frequency
Write	Sitting to write	Sitting in front of a desk to write or use a computer
	Standing to write	Standing in front of a desk to write or use a computer
Look	Looking straight forward	Gazing directly at a target near the eyeline
	Looking up	Looking up toward the ceiling or otherwise above the eyeline
	Looking down	Looking downward, generally to read or use a cell phone, etc.
Sleep	Sleeping face-down	Closing eyes, folding hands, resting head on hands
Listen	Erect ears	Ears are oriented forward to listen to a speaker
	Looking at speaker	Looking at the speaker while listening in an effort to understand clearly
Explain	Upright speaking	Posturing with feet flat on the ground to speak
	Standing to speak	Standing still to speak
	Speaking with activity	Utilizing gestures while speaking to express emotion or emphasize tone
Hand raise	Raising left hand	Raising the left hand
	Raising right hand	Raising the right hand
Clearing the blackboard	Wiping down	Holding a blackboard eraser in one hand and wiping it across the blackboard
Drinking water	Drinking water	Lifting a drinking vessel to the mouth
Using computer	Using computer	Turning on the computer screen, utilizing keyboard and mouse with hands, focusing eyes on the screen
Getting up	Getting up	Changing from a sitting position to standing and walking

**Figure 6:**
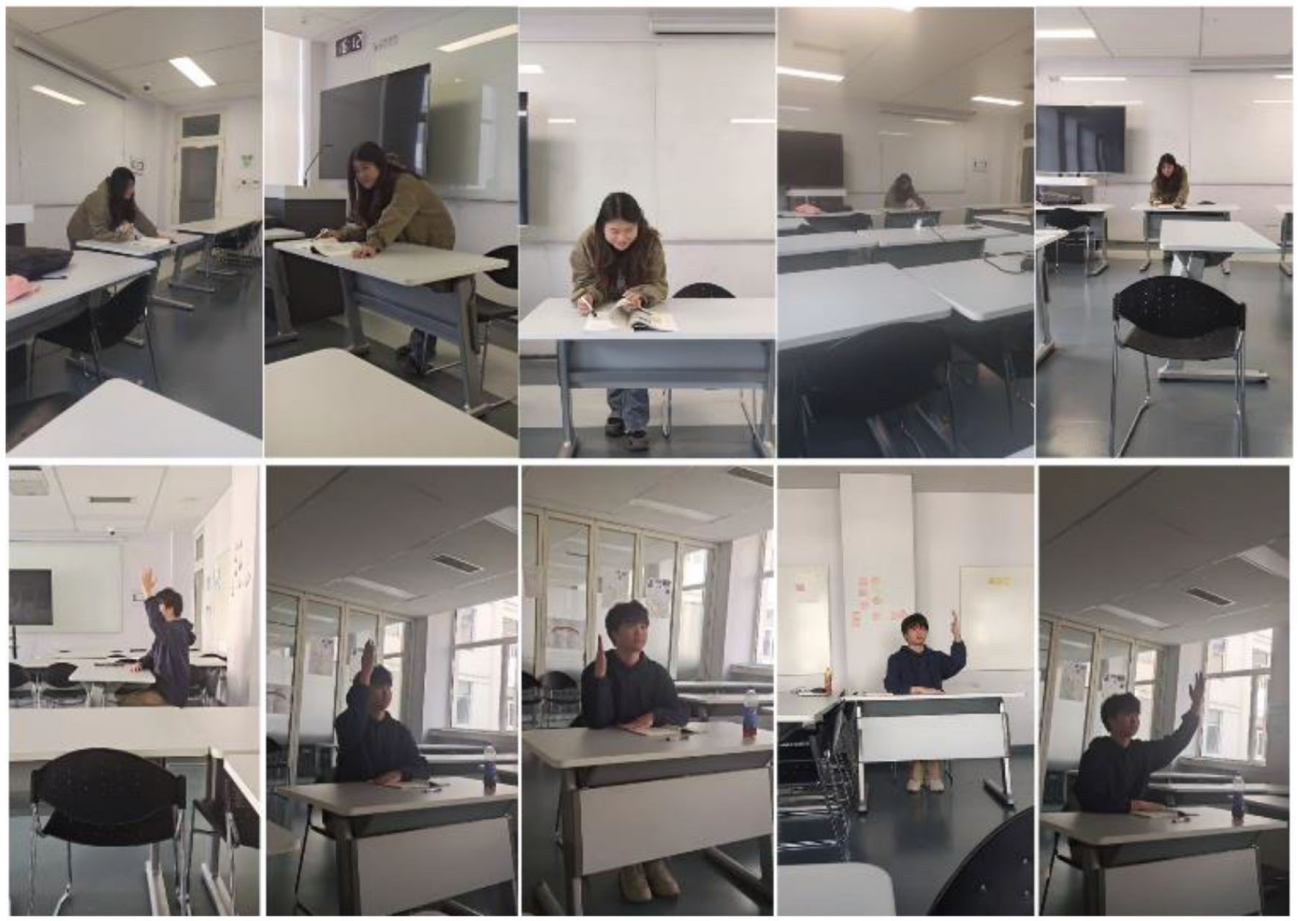
Screenshots of videos taken from different angles and different scales.

The human body is roughly symmetrical, so the angle of view can be divided into three main types. When the body plane aligns perpendicularly to the observation direction, it constitutes a positive viewing angle. An oblique angle results when the body plane forms an angle with the observation direction. A side-view angle is established when the body plane runs parallel to the observation direction. Accommodating these varying angles required a simultaneous consideration of different scales, as illustrated through the screenshots of our videos shown in Figure 6.

## 5. Experiment

### 5.1. Dataset

To test the performance of the proposed method, we conducted experiments on the IsoGD dataset and the self-made EuClass dataset. The IsoGD dataset contains the depth and RGB data of 249 gestures, each containing 47,933 labeled video samples; each sample length is between 9 and 405 frames and the resolution is 320^*^240. The EuClass classroom behavior dataset contains data for 13 behavior types of teachers and students in the classroom environment. The video resolution is 720^*^1,280 and the length of each sample is between 30 and 540 frames. The dataset was created by taking videos of two people performing various behaviors relevant to daily in-class learning, with the shooting angle based on the human body's medial axis from the left, right, and front angles. The dataset was designed to be multi-view and multi-scale by using both long and close shooting distances.

### 5.2. Experimental details

The proposed method was implemented using PyTorch on an NVIDIA V100 GPU. Drawing from the performance characteristics of the IsoGD dataset described in a previous study (Chen et al., 2022), we used the I3D network and the sampling length used by IsoGD. During training, each frame was randomly cropped to 224 × 224 while during inference, the center of the frame was maintained at the same size. Stochastic gradient descent was applied to optimize the neural network parameters. The initial learning rate was set to 0.1, the number of processes to 1, and the momentum to 0.9; the batch size was set to 8 during training and to 10 during testing. The training phase spanned 30 epochs.

### 5.3. Comparison with state-of-the-art methods on IsoGD dataset

We tested the proposed method on the RGB and depth data of the IsoGD dataset in comparison against other state-of-the-art methods, which were also operated on the single modality data of RGB/depth. Note that as the code of some of the methods is not released, we give the performance with our reproduction.

As shown in Tables 2, 3, the proposed method performed well in terms of both RGB and depth. On RGB modality data, it outperformed the second-best method by 0.84%. The second-best method uses I3D as the backbone network, combining local and dual attention mechanisms. The proposed method includes a two-level attention mechanism for analyzing similar behavioral video data. The attention mechanism was designed to focus on relevant features in videos based on different perspectives and scales, which improves the recognition accuracy; the intra-class differential representation learning process enhances overall accuracy. On depth modality data, our network improves the accuracy of identification by 1.16% compared to the second-best method.

**Table 2:** Comparison of IsoGD (RGB/depth) with state-of-the-art methods.

**Method**	**Modality**	**Acc (%)**
Li et al. (2018)	RGB	37.28
Miao et al. (2017)	RGB	45.07
Duan et al. (2018)	RGB	46.08
Li et al. (2019b)	RGB	46.12
Zhang et al. (2017)	RGB	51.31
Zhang et al. (2018)	RGB	55.98
Lin et al. (2018)	RGB	56.21
Zhu et al. (2019)	RGB	57.42
Zhou et al. (2021)	RGB	62.66
Chen et al. (2022)	RGB	62.73
Proposed	RGB	63.57

**Table 3:** Comparison of IsoGD (Depth) with state-of-the-art methods.

**Method**	**Modality**	**Acc (%)**
Li et al. (2018)	Depth	40.49
Miao et al. (2017)	Depth	48.44
Li et al. (2019b)	Depth	49.01
Zhang et al. (2017)	Depth	49.81
Zhang et al. (2018)	Depth	53.28
Zhu et al. (2019)	Depth	54.18
Duan et al. (2018)	Depth	54.95
Lin et al. (2018)	Depth	56.35
Zhou et al. (2021)	Depth	60.66
Chen et al. (2022)	Depth	61.72
Proposed	Depth	62.88

### 5.4. Comparison with other methods on self-made EuClass dataset

The shooting dataset encompasses a large amount of multi-view and multi-scale video data. We experimented on EuClass with a differentiated representation learning method for similar behaviors and achieved an accuracy of 91.03%. We compared the results of the proposed method against those of other methods (Table 4) to find that performs 0.9% better than the I3D network, 10.78% better than the 3D network CNN, and 5.67% better than Res C3D.

**Table 4:** Recognition accuracy of dataset EuClass using different methods.

**Method**	**Acc (%)**
Li et al. (2018)	80.25
Miao et al. (2017)	85.36
Li et al. (2019b)	86.06
Yuan et al. (2019)	88.25
I3D (Carreira and Zisserman, 2017)	90.13
Li et al. (2023b)	91.00
Proposed	91.03

### 5.5. Ablation experiment

We conducted ablation experiments to validate the efficacy of the proposed method. We utilized the I3D network as a baseline to discern the performance of each component. By incorporating improved elements step-by-step, we assessed the performance of RGB modal data from both IsoGD and EuClass datasets. The progressive inclusion of the two-level attention mechanism and intra-class differential representation module is outlined in Table 5. Notably, the integration of the intra-class differential representation learning module for similar behaviors led to a 2.29% accuracy boost on the IsoGD dataset when compared to the baseline. For the EuClass dataset, the recognition accuracy was comparable to that of the baseline, with a 0.9% enhancement. This improvement can be primarily attributed to the intra-class differential representation learning of similar behaviors, which effectively mitigates the impact of different viewing angles and different scales on accuracy.

**Table 5:** Ablation experiment results.

**DataSet**	**Method**	**Acc (%)**
IsoGD (RGB)	I3D	61.28
	I3D+TL—Attention	62.73
	TL-Attention+MSE	63.57
EuClass	I3D	90.13
	TL-Attention	90.58
	TL-Attention+MSE	91.03

Unlike the baseline I3D method, the proposed method also includes a two-level attention module focused on channel and spatial features. For problems with different perspectives and scales in real scenes, the intra-class differential representation module can be applied to extract data features for the same behavior type and perform similarity comparisons, enhancing learning accuracy.

## 6. Conclusions

In this study, we developed a 3D convolutional network-based two-level attention module and an intra-class differential representation learning module for recognizing similar behaviors. We evaluated the proposed approach the IsoGD and a self-made dataset, EuClass. The backbone network employs the I3D architecture, augmented with a two-level attention module for spatial and channel feature extraction. By processing pairs of videos featuring identical behaviors but with distinct representations, the network performs feature extraction followed by classification through intra-class differential representation learning.

The proposed method achieved 63.57% accuracy on the RGB modality of the IsoGD dataset, surpassing the baseline accuracy of I3D while outperforming current state-of-the-art approaches. An accuracy of 91.03% was achieved on the EuClass dataset, which is higher than the baseline accuracy of I3D. These results demonstrate the effectiveness of the proposed model in addressing the challenges posed by multi-view and multi-scale data in behavior recognition tasks.

## Data availability statement

The original contributions presented in the study are included in the article/supplementary material, further inquiries can be directed to the corresponding author.

## Ethics statement

Written informed consent was obtained from the individual(s) for the publication of any potentially identifiable images or data included in this article.

## Author contributions

DZ: Conceptualization, Methodology, Validation, Writing—original draft. CC: Writing—original draft. FT: Data curation, Writing—review and editing. BQ: Data curation, Writing—review and editing. WL: Writing—review and editing, Data curation. XH: Writing—review and editing. SL: Writing—review and editing.
